# Is music enriching for group-housed captive chimpanzees (*Pan troglodytes*)?

**DOI:** 10.1371/journal.pone.0172672

**Published:** 2017-03-29

**Authors:** Emma K. Wallace, Drew Altschul, Karoline Körfer, Benjamin Benti, Amanda Kaeser, Susan Lambeth, Bridget M. Waller, Katie E. Slocombe

**Affiliations:** 1 Department of Psychology, University of York, York, United Kingdom; 2 Department of Psychology, University of Edinburgh, Edinburgh, United Kingdom; 3 Department of Veterinary Medicine and Surgery, University of Texas MD Anderson Cancer Center, Houston, Texas, United States of America; 4 Department of Psychology, University of Portsmouth, Portsmouth, United Kingdom; University of Zurich, SWITZERLAND

## Abstract

Many facilities that house captive primates play music for animal enrichment or for caregiver enjoyment. However, the impact on primates is unknown as previous studies have been inconclusive. We conducted three studies with zoo-housed chimpanzees (*Pan troglodytes*) and one with group-housed chimpanzees at the National Centre for Chimpanzee Care to investigate the effects of classical and pop/rock music on various variables that may be indicative of increased welfare. Study one compared the behaviour and use of space of 18 animals when silence, classical or pop/rock music was played into one of several indoor areas. Overall, chimpanzees did not actively avoid the area when music was playing but were more likely to exit the area when songs with higher beats per minute were broadcast. Chimpanzees showed significantly fewer active social behaviours when music, rather than silence, was playing. They also tended to be more active and engage in less abnormal behaviour during the music but there was no change to either self-grooming or aggression between music and silent conditions. The genre of music had no differential effects on the chimpanzees’ use of space and behaviour. In the second study, continuous focal observations were carried out on three individuals with relatively high levels of abnormal behaviour. No differences in behaviour between music and silence periods were found in any of the individuals. The final two studies used devices that allowed chimpanzees to choose if they wanted to listen to music of various types or silence. Both studies showed that there were no persistent preferences for any type of music or silence. When taken together, our results do not suggest music is enriching for group-housed captive chimpanzees, but they also do not suggest that music has a negative effect on welfare.

## Introduction

Ensuring good welfare for captive animals, such as chimpanzees, is important not only to maximise the psychological wellbeing of the animals, but also to improve the quality and validity of research conducted in captive settings, and to maximise education opportunities in zoos [1]. Environmental enrichment is a commonly used method for improving animal welfare. A major goal of enrichment is to simulate activities of their wild counterparts and encourage species-typical behaviours [2]. Successful enrichment often entails encouraging greater diversity of behaviours [3] and more positive active behaviours, such as foraging for food. One inexpensive, durable form of potential enrichment is auditory enrichment and a 2006 European Directive recommends its use with laboratory primates [4].

One method of assessing the success of a form of enrichment is how much the target animals use it. However, we feel that for the purpose of these studies in order to be able to classify music as a form of auditory enrichment it must be seen to have a positive effect on animal welfare by reducing negative or abnormal behaviours whilst increasing positive forms of activity. Wild chimpanzees display high levels of aggression[5, 6], which, if unmanaged, can be problematic in captivity as limited enclosure space means that animals cannot always escape their aggressors, making serious injury more likely. Therefore, enrichment should not cause levels of aggression to increase. If, however, reductions in aggression and abnormal behaviours that are commonly observed in chimpanzees [7] are coupled with an overall decrease in activity, this may not be indicative of a positive welfare change. Freezing behaviour and reduction in activity may be associated with helplessness, which is where an individual has no expectation of a relationship between responses and outcomes [8] and such a state is associated with high levels of anxiety [9]. Thus overall reductions in activity in the face of environmental factors out of the animals’ controls could be indicative of anxiety and helplessness. For an animal to be deemed to have good welfare the presence of positive behaviours is as important as the absence of negative ones [10]. Therefore, an increase in active social behaviours, such as playing and grooming, as well as a reduction of inactivity, aggression and abnormal behaviours would indicate that music is enriching.

Music has been reported to be enriching for laboratory-housed chimpanzees, with one study finding that five ‘relaxing’ genres of music (classical, country, ethnic, oldies and soft) together reduced aggression, abnormal behaviours and increased social grooming [11]. However, these positive changes were also coupled with an increase in inactive behaviours. In this study, the chimpanzee could not choose to avoid the music, therefore, it is possible that the animals were responding to the music in a helpless manner. Another study [12] also found that music increased social interactions and decreased aggression in laboratory chimpanzees but rather than looking at differences in genre of music, whether or not human vocals were present in the music was manipulated. From this, they found that music solely comprised of instruments had a greater effect on increasing social interactions, whereas music including human vocals, especially that with slower tempos (50 to 90 beats per minute) was better at reducing aggression.

The results of these two studies [11, 12] suggest that music could have an enriching effect on chimpanzees, however, in both of these studies the animals were not given the option to avoid the music, meaning the observed changes in behaviour could have been part of a coping strategy for this uncontrollable situation. Other studies have given animals the option to choose what they want to listen to. A study that gave marmosets and tamarins *(Callithrix jacchus* and *Saguinus oedipus)* choice over what they could listen to found that they preferred slow tempo lullabies over very fast tempo techno music and preferred silence over the lullabies [13]. Two out of three orangutans (*Pongo pygmaeus*) at Toronto Zoo chose to listen to silence over seven different genres of music, including Tuva throat singing, which was included in the study as it is considered the form of music that most closely resembled orangutan long calls [14]. Both of these studies show that when primates are provided with the ability to control what they can hear, they choose silence or no music, suggesting that music is not enriching.

To date, music as enrichment has been studied with gorillas (*Gorilla gorilla gorilla*) [15, 16], orangutans [14] and gibbons (*Hylobates moloch*)[17] in zoos but chimpanzees have only been studied in a laboratory setting [11, 12].

In this study, we aimed to assess the impact of music on the behaviour of captive chimpanzees whilst giving them the option to avoid the music if they desired. We also directly compared the effects of classical music with contemporary Pop/rock music, the two genres of music which animals are most likely to be exposed to inadvertently through music played for the enjoyment of care staff. In addition, we gave the chimpanzees the ability to control whether they could hear different genres of music or silence. Studies 1, 2 and 3b were conducted with 18 chimpanzees at Edinburgh Zoo and study 3a was conducted with 38 chimpanzees at the National Center for Chimpanzee Care in Bastrop, Texas. The relatively large zoo sample and the use of similar paradigms across two sites mean our study has the potential to generate representative and generalizable results.

### Statistical analysis

All statistical tests are reported as two-tailed tests, with the alpha level set at 0.05. In order to assess whether data were normally distributed and thus suitable for parametric tests, the data were visualised and Shapiro-Wilk normality tests were run. All tests were run using SPSS v.21.

## Study 1

### Aims and research questions

This study aimed to examine if the presence of music affected the chimpanzees’ use of space and their general behaviour. In contrast to previous studies, the enclosure design at Edinburgh Zoo meant that if music was played into just one area, it was possible for individuals to avoid the music in one area while the music was played in another area if they chose to do so.

We aimed to address the following questions: (i) Does the presence of music in part of the enclosure affect the animals’ use of space; do they approach or avoid the area where music is playing? (ii) Does music affect the behaviour of the individuals exposed to the music? We predicted that if music was having a positive impact on welfare we would find increases in social and active behaviours combined with decreases in aggression and abnormal or stress related behaviours; and (iii) Do classical music and pop/rock music have differing effects on the use of space and behaviour of the animals? Previous studies have shown that instrumental classical music reduces aggression and increases social grooming in laboratory chimpanzees [12] suggesting that the classical music in this study may have positive effects.

## Methods

### Ethics statement

Studies 1, 2 and 3b were approved by the University of York regulated Department of Biology Ethics Committee and Edinburgh Zoo, part of the Royal Zoological Society of Scotland (RZSS).

### Study site

Research was undertaken at Budongo Trail, Edinburgh Zoo, Scotland. The facility was built in 2008 and has capacity for 40 chimpanzees. The facility is over 1500m^2^ and comprises of three indoor ‘pods’, an off-show bedding area and an outdoor enclosure, all linked by tunnels. Each of the ‘pods’ and the outdoor area contain large, wooden climbing structures with built-in metal baskets that can be used for day beds, encouraging natural bedding behaviours. This layout allows the animals to choose their locations and social proximity to other group members and to split into sub-groups that vary in composition of individuals, allowing their natural fission-fusion social system to be expressed.

### Participants

During the study period there were 18 adult chimpanzees (10 females and eight males; S1 Table). The group was comprised of individuals originally either from Edinburgh Zoo or Beekes-Bergen Safari Park who were integrated into the Edinburgh group in 2010 [18]. Before living in the safari park, these animals were housed in an experimental laboratory. None of the 18 animals had been exposed to music since 2010. Prior to 2010 it is believed that all Edinburgh Zoo individuals heard music played for caretakers but the music exposure history of the Beekes-Bergen individuals was unknown.

### Materials

Music was played using an Ipod Nano^®^ and an Anchor Liberty minivox battery powered speaker. Music was played into one target pod through open mesh areas in the keeper’s doors at a height of approximately 1.5m. To ensure the majority of the sound was channelled into the chosen pod and that as little noise as possible was heard in other areas of the enclosure, music was broadcast from a speaker housed in an insulated box (S1 Fig). Sound levels were set so that no music could be heard in at least one of the other indoor pods and it was audible at a comfortable level for human experimenters at all points in the target pod. Data were commentated in real time onto an Olympus DM650 dictaphone and transcribed later using Olympus Sonority software.

### Stimuli

Since the music history of the chimpanzees is unknown prior to 2010, the songs used for the pop/rock music were those released into the charts from 2010 onwards to ensure they were novel to all animals. Classical instrumental music with between 50 and 90 beats per minute (BPM) has been shown to increase social grooming in laboratory chimpanzees [12] and was therefore used for this study. Music without dramatic passages was chosen to increase potential for the music to have a calming effect. As most contemporary pop/rock music is much faster than classical music, songs with greater than 90 BPM were chosen to replicate radio music for keepers/care givers use when preparing food, cleaning enclosures etc.

Fifteen pieces of music were selected: seven classical pieces and eight pop/rock songs (S2 Table). One piece of music followed immediately after the previous one finished. The running time of the classical playlist was 30 minutes and 23 seconds and the pop/rock playlist lasted 30 minutes and 2 seconds. Music was equalised in overall amplitude using Audacity auditory editing software. Each piece of music was brought to an average amplitude by reducing the volume of loud passages and increasing the volume of quieter ones. For each type of music, three playlists were created with each version having a different order (S3 Table). This was done so the chimpanzees did not habituate to the stimuli or display anticipatory avoidance behaviour towards certain songs. For each genre of music the three playlists were played six times with the exception of the first classical and pop/rock lists that were each played seven times.

### Data collection

Data were collected over 14 weeks (April-May; August–September 2013). Four experiments were conducted each week on two separate days between 12:00–13:00 and 14:15–15:15. In total there were 38 hour-long trials; 19 where music was played into pod two (9x classical music and 10x pop/rock) and 19 where music was played into pod one (10x classical music and 9x pop/rock). The order of music and silence were counterbalanced across trials with music occurring in the first 30 minutes of the trial 19 times. Observations were undertaken in the public viewing area of the enclosure to minimise the effect of collecting data on the behaviour of the chimpanzees.

Instantaneous scan samples (recording data at a specific point in time) [19] were taken recording the identity and behaviour of each individual present in the target pod (Table 1). During each condition there were 11 scans per condition; 10 with an inter-scan interval of three minutes, and a final scan that occurred two minutes after the tenth scan. The 13 behaviours were recorded during scan samples based on previous research on chimpanzee welfare using behavioural indicators [20, 21, 22, 23]. The 13 behaviours were collapsed down into five behavioural categories; active, passive, socially active, self-grooming and abnormal (Table 1). In addition to the scan samples, all occurrence data [19]on exits and entrances from the experimental pod were recorded as well as all aggressive events (displaying, chasing and/or hitting another individual) within the target pod.

**Table 1:** Behaviours recorded in instantaneous scan samples.

*Behaviour category*	*Behaviour*	*Description*
Passive	Resting	Resting when standing, sitting or lying
Active	Travel	Walking or running
	Climbing	Travelling in an upwards trajectory
	Foraging	Moving whilst looking for or handling food
	Eating	Consuming food
Social Active	Playing	Interacting with another individual or an object in a playful manner
	Grooming another	Manipulating the hair on another’s body
	Receiving grooming	Having hair manipulated by another
	Mutual grooming	Two individuals manipulating the hair on the other conspecific’s body
Self-grooming	Self-grooming	Manipulating the hair on own body
Abnormal	Abnormal and stress related behaviours	Any abnormal behaviour indicative of stress: regurgitation and reingestion (R/R), urine drinking, faeces eating, plucking fur, scratching and yawning
Not included	Aggression Other	Displaying, chasing or physical contact in an aggressive manner Anything else not mentioned above; exact details noted

### Data analysis

For data that was analysed for this study as well as studies 2, 3a and 3b, please see S1 Dataset.

#### Do the chimpanzees approach or avoid the target pod where music is playing?

The time each individual spent in the music pod for each of the music and silence conditions within a trial was calculated from their entry and exit times. If an individual spent multiple periods in the pod, a mean duration spent in the target pod during each of the silence and music periods was calculated. The minimum requirement for a trial to be included in the analysis for a particular individual was that the individual had to be present for at least three minutes in each of the two (music and silence) conditions. The mean duration each animal spent in the target pod during the silence and music periods from all its eligible trials was then calculated. All individuals were present in at least 2 eligible trials (range 2–21) resulting in N = 18.

#### Does BPM of the music affect the chimpanzees’ use of space?

To see if the BPM of certain songs across the classical and pop/rock genres had different effects on chimpanzees’ use of space, the song playing as an individual entered and/or exited the music pod was identified. The criterion for an individual to contribute data to this analysis was that an individual had to enter and exit the pod at least five times during music periods, resulting in N = 18. The mean BPM from all of an individual’s exit or entry songs (S2 Table) was then calculated.

#### Does music affect the behaviour of the individuals exposed to the music?

Data from the instantaneous scan samples were used to examine active, passive, socially active, self-grooming and abnormal behaviour in music and silent periods. For each individual we only included data from ‘eligible’ trials where individuals were present at for at least one scan in each of the music and the silence periods so we could examine differences between these matched periods. This helped to control for inter-day differences in the behaviour of the chimpanzees due to changes in group dynamics or external factors, such as fluctuations in visitor numbers or building maintenance being undertaken.

Separate analyses were run for each of the five behaviour categories (active, passive, socially active, self-grooming and abnormal). Across eligible trials, the number of scans in which an individual demonstrated a behaviour category (e.g. active) was divided by the number of scans he/she was present in that condition (music / silence) to create proportion measures. To enter this analysis an individual’s proportions had to be based on data from a minimum of two trials, resulting in N = 18.

The total number of aggressive events where an individual was acting as an aggressor in music and silent periods across trials was divided by the total time that an individual was present in the associated condition (taken from entry/exit times). This then gave the rates of aggression per individual per hour in the target pod during music and silence. Only individuals who were observed acting as the aggressor at least once (N = 11) were included in this analysis.

#### Do classical music and pop/rock music have differing effects on the use of space and behaviour of the animals?

To compare the effects of classical and pop/rock music on duration in the music pod, rates of aggression and proportion of scans engaged in active, passive, socially active, self-grooming and abnormal behaviours, difference values (total or mean value from one genre minus the total or mean value from the matched silence periods) were created. The criterion for entry into the “classical difference” and “pop/rock difference” analyses was being present for at least one scan or 3 minutes duration in both the music and silence periods of a single experimental trial, for a minimum of two classical trials and two pop/rock trials, resulting in N = 16 individuals.

### Statistical analyses

Paired T-tests were conducted to test for differences between music and silence conditions and to test between”classical difference” and”pop/rock difference”. Effect sizes (*d*) were calculated using an online tool (http://www.cognitiveflexibility.org/effectsize/), whilst the sample sizes that post-hoc power analyses indicated would be required to reach significance were calculated using G*Power 3.1.9.2. When using Cohen’s *d* as an effect size, a large effect would be considered 0.80 and above, a medium sized effect would be 0.50 and 0.20 would be a small effect [24].

## Results

### Do the chimpanzees approach or avoid the target pod where music is playing?

The chimpanzees (N = 18) showed no significant difference in the amount of time they spent in the pod when music was playing (mean = 914s, SD 341) compared to when the pod was silent (mean = 975s, SD 191; Paired t-test T(17) = -1.11 p = .280; *d* = 0.22). The effect size of 0.22 was small and post-hoc power analyses indicated that for such a small effect to become significant we would have needed to have tested 225 individuals.

### Does BPM of the music affect the chimpanzees’ use of space?

**Fig 1:**
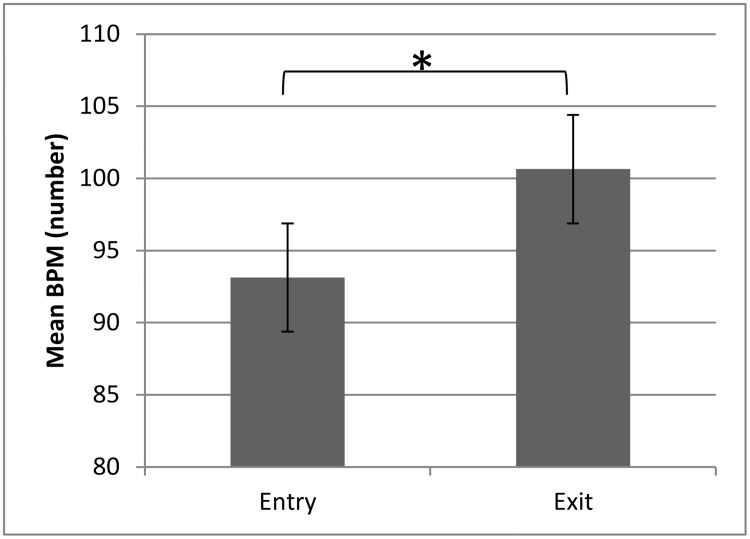
Mean BPM of songs playing when chimpanzees entered and exited the pod with the music playing. Error bars represent standard error. *P < .05

The mean BPM of the music playing when the chimpanzees (N = 18) entered the music pod was significantly lower than the BPM of the songs they exited to (Paired t-test T(17) = -2.23, p = .039; *d* = 0.04; Fig 1).

### Does music affect the behaviour of the individuals exposed to the music?

There was no difference in the proportion of time chimpanzees spent being passive or self-grooming between music and silence conditions but there were trends for chimpanzees showing less abnormal behaviours when the music was playing and more active behaviours during the music. The chimpanzees also displayed significantly fewer socially active behaviours whilst the music was broadcast (Table 2).

**Table 2:** Results for paired T-tests. Tests were comparing mean proportion of scans spent engaging in Passive, Active, Socially Active, Self-grooming and Abnormal Behaviours between Music and Silence trials. Trends are italicised and significant differences are shown in bold and underlined.

Behaviour	Mean proportion of music scans engaged in the behaviour category (SD)	Mean proportion of silence scans engaged in the behaviour category (SD)	T value (df = 17)	p value	*d*	Sample size power analyses indicated would be required to reach significance
Passive	62.70 (17.50)	63.29 (15.31)	-0.10	.920	0.04	6766
*Active*	*12*.*94 (8*.*44)*	*9*.*94 (6*.*63)*	*2*.*02*	.*059*	*0*.*28*	*168*
**Social Active**	**8.00 (7.49)**	**16.33 (10.69)**	**-5.05**	**< .001**	**0.91**	**N/A**
Self-grooming	4.83 (4.62)	5.39 (3.90)	-0.73	.477	0.09	1607
*Abnormal Behaviours*	*0*.*65 (1*.*28)*	*1*.*35 (1*.*80)*	*-1*.*88*	.*080*	*0*.*33*	*122*

Rates of aggression of the chimpanzees (N = 11) were not significantly different between when music was playing (mean = 0.78/hr, SD 0.85) and when there was silence (mean = 0.26/hr, SD 0.48; Paired t-test T(10) = 1.72, p = .115; *d* = 0.57).

### Do classical music and pop/rock music have differing effects on the use of space and behaviour of the animals?

There were no significant differences in the chimpanzees’ (N = 16) duration spent in the music pod, the proportion of scans engaged in self-grooming, active, socially active, passive and abnormal behaviours between classical and pop/rock music (Table 3) but there was a trend for the chimpanzees showing a higher rate of aggression during music compared to matched silence periods when pop/rock music (Mean = 0.53 SD 1.09) was being played compared to classical (Mean = -0.02 SD 0.62). However, post-hoc Paired T-test found that rates of aggression were not significantly higher during the pop/rock music (Mean = 1.88 SD 4.16) than during the associated silence periods (Mean = 1.06 SD 2.74; Paired t-test T(15) = -1.77, p = .097; Bonferroni corrected alpha value = .025).

**Table 3:** Results comparing “classical difference” with “pop/rock difference”. Positive mean values indicate more of the behaviour was observed in the music period (classical or pop/rock) compared to the matched silence period; whilst negative mean values indicate more of the behaviour was observed in the silence period compared to the matched music period (classical or pop/rock).

Type of Data Analysis	Mean “classical difference” from matched silence periods (SD)	Mean “pop/rock difference” from matched silence periods (SD)	T value (df = 15)	p value	*d*	Sample size power analyses indicated would be required to reach significance
Duration	-31 seconds (148)	5 seconds (193)	-0.54	.598	0.14	665
Passivity	5.08 (19.44)	-5.10 (12.95)	1.55	.143	0.39	88
Activity	1.72 (5.18)	1.28 (5.03)	0.23	.817	0.06	3612
Social Activity	-4.39 (5.12)	-3.94 (5.01)	-0.26	.800	0.06	3612
Self-grooming	-1.00 (3.01)	0.44 (2.83)	-1.26	.225	0.30	147
Abnormal Behaviours	-0.45 (2.03)	-1.20 (3.42)	0.66	.522	0.17	452
Aggression	-0.02/hour (0.64)	0.53/hour (1.12)	-1.68	.113	0.43	73

## Discussion

The chimpanzees seemed to show little reaction to music generally. Individuals spent similar amounts of time in the target pod regardless of whether or not music was playing. This suggests that the animals did not actively seek out the music but equally they were not trying to avoid it. We did, however, find that the music the chimpanzees entered the pod to had a significantly lower number of BPM than the music they exited to. This suggests that they may show a ‘preference’ for music with lower BPM. This supports Videan et al.’s [12] findings that music with lower BPM had more positive effects on laboratory chimpanzees, in this case in terms of reducing aggression. Manipulation of the tempo of the same pieces of music may be an effective way to further test to effect of tempo on chimpanzees’ use of space or behaviour.

When considering both genres of music together, significantly fewer socially active behaviours (playing and grooming) were displayed by the chimpanzees when the music was playing compared to when there was silence. As mentioned above, in these studies we have chosen to consider something as enriching if the target animals display positive welfare changes, such as an increase in social behaviours. Our finding that the chimpanzees showed less play and grooming behaviours during the music strongly suggests a lack of enriching effect. It also contrasts with Howell et al. [11] who found music increased social grooming. We also found a trend towards an increase in active behaviours, whereas Howell et al. [11] found an increase in inactive behaviours. This could suggest that the chimpanzees in our study were not actively trying to avoid the target music pod when the music was playing because the cost of avoiding a preferred pod, being in proximity to a preferred individual etc., may have been too high. Instead they may have tried to find areas within the same pod where they could not hear the music or the volume was not as great, which lead to an increase in their activity.

Additionally, we found a trend towards music reducing abnormal behaviours. Although our effect size is small, similar results were found by Wells et al. [15]. They found a trend towards a reduction in what they termed abnormal behaviours, when a group of gorillas were exposed to classical music. However, the constituent behaviours that made up their category of abnormal behaviours did not include regurgitation and reingestion as in our study. By contrast, Robbins and Margulis [16] reported that both classical and rock music tended to increase the prevalence of regurgitation and reingestion in their three gorillas, as well as hair plucking and stereotypical locomotion. It is likely that the sampling technique used in our study was not optimal for detecting differences in abnormal behaviours, which can happen very quickly and be quite subtle. We address this possibility in study two, which aimed to explore the effect of music on abnormal behaviour in more detail.

## Study 2

### Aim and research questions

This study aimed to examine the effect of music on rarer, abnormal behaviours that may have been missed in study one due to instantaneous scan sampling [19]. In this study we employed continuous focal sampling [19], which is where the behaviour of an individual is continually recorded. This is a more sensitive method for observing abnormal behaviours and meant we were able to calculate exact durations engaged in each type of behaviour.

We aimed to address the following questions: First, does the presence of music increase or decrease abnormal behaviour rates in focal individuals compared to matched silent periods? Based on the results of study 1, we predicted that music would lead to a decrease in abnormal behaviours. Second, do classical music and pop/rock music have differing effects on the rate of focal animals’ abnormal behaviour?

### Methods

#### Participants

For this study, we focused on three individuals: Rene, Paul and Lianne. These individuals were chosen as long-term behavioural data showed that they displayed the highest rates of abnormal behaviours, making them ideal candidates in which to examine any effects of music on abnormal behaviours.

#### Stimuli

The same music and playlists were played into pods one and three simultaneously using two sets of the materials used in Study 1. This was done in order to increase the chances of the focal individual hearing the music, whilst also providing areas without music so that it could be avoided. Data was dictated and transcribed as in Study 1.

#### Data collection

Data were collected from January to May 2014. Before undertaking data collection, *A Priori* power analyses were run, which determined that with a power of 0.8, to obtain an effect size of 0.5 would require 34 trials for each individual. Whilst we conducted 37 trials for Lianne, logistical constraints meant we only ran 26 trials for Rene and 29 for Paul (total 92 trials). The first 30 minutes was a control silence period, followed by 30 minutes of music and then a second 30 minutes of silence. Continuous focal sampling [19] was used for a period of 90 minutes, with the start and end times of each behaviour (Table 1) recorded so exact durations could be calculated.

As individuals could choose to avoid the music, in some trials the focal individual was not exposed to music during the music period. We only included data from the trials when the focal individuals were actually exposed to music (present in pod 1 or 3) for at least five minutes during the 30 minute period when music was played. Using this criteria resulted in 12 trials (7 from Paul, 1 from Lianne and 4 from Rene) being removed from the dataset, leaving a total of 80 trials (36 for Lianne and 22 for both Rene and Paul). Of these 80 trials, the type of music played was either pop/rock (37; 12 for Rene, 9 for Paul and 16 for Lianne) or classical (43; 10 trials for Rene, 13 for Paul and 20 for Lianne).

If individuals were observed for much longer in one condition than another, they would have had more opportunity to display a wider variety of behaviour in the condition with more observation time. To counter this potential problem and to ensure that we were comparing similar time periods across music and silent conditions, a random number generator (www.random.org) was used to select which of the two silence periods would be compared to the music period from that trial. Secondly, we then compared the observation time in the matched silence and music periods and found that the mean duration of observation in silence periods fell within 1 SD of the mean duration of observation in the music periods (S4 Table), and so were comparable.

## Data analysis

Data for each of the three individuals was analysed separately. The duration an individual spent engaged in abnormal behaviours during each condition was divided by the observed duration in that condition (e.g. excluding any out of sight periods; in music periods only time spent in the pods where music was playing so the individual was exposed to music). This resulted in a percentage of available time spent engaged in abnormal behaviours being calculated for each silence and music period. To investigate the effect of the different genres of music on behaviour, we created difference values as used in Study 1 (see Data Analysis).

### Statistical analysis

All data met the assumptions of parametric testing and data for each individual was analysed separately. To compare the effects of music and silence (matched pairs from each trial) on behaviour, paired T-tests were used and to compare the effects of “pop/rock difference” with “classical difference” on behaviour, independent samples T-tests were used as classical and pop/rock conditions were broadcast during different trials.

## Results

There were no significant differences found in any of the individuals for any abnormal behaviour between when music was playing and when there was silence or between “classical difference” and “pop/rock difference” (Table 4), possibly due to the fact that abnormal behaviours were displayed at low levels for all three individuals (Table 5).

**Table 4:** Results comparing the percentage time spent displaying abnormal behaviours between music and silence. Paired T-tests were used to compare Music with Silence and “classical difference” with “pop/rock difference” were compared with Independent T-tests for Rene (N = 22), Lianne (N = 36) and Paul (N = 22).

Individual (N)	Music vs Silence T value (df = 21)	p value	Effect Sizes	Pop/rock Difference vs Classical Difference T value (df = 20)	p value	Effect Sizes
Rene (N = 22)	-0.56	.585	0.12	1.67	.110	0.74
Lianne (N = 36)	-0.41	.684	0.10	-1.30	.203	0.18
Paul (N = 22)	-0.01	.994	0.001	1.10	.286	0.51

**Table 5:** Percentage time spent displaying abnormal behaviours during music and silence periods for all three individuals.

Individual	Music (SD)	Silence (SD)
Rene	2.57 (5.37)	3.52 (6.38)
Lianne	8.34 (14.32)	10.07 (17.06)
Paul	4.11 (7.88)	4.12 (4.22)

## Discussion

The results from study 1 suggested that music might decrease rates of abnormal behaviours, however, the results of study 2 do not support this view. As continuous focal sampling was used in study 2, all instances of abnormal behaviour were recorded rather than just those that occurred at the point of a scan sample. Additionally, we had a larger number of trials than in study 1 and focussed on individuals with relatively high baseline rates of abnormal behaviour. This means that we were able to accurately see if music was having a specific effect on abnormal behaviours. As music compared to silence generated no significant effects and small effect sizes, we can be relatively confident that overall music was not having an effect on rates of abnormal behaviours in those most prone to displaying them in this group.

Data have been analysed thoroughly in an attempt to find any effects of music or genre on the individuals’ behaviour. Running a large number of statistical tests may have increased chances of finding Type 1 errors but given the lack of significant results this does not affect the interpretation of our data. It is, perhaps a greater concern that our great number of null results may be a result of insufficient statistical power and represent type 2 errors. However, the small effect sizes that accompanied most non-significant results indicates that the music is having minimal effect on behaviour and even with a larger number of trials, we would likely not have found any significant differences.

Overall, this study suggests that both classical and pop/rock music have no positive or negative effect on the behaviour of three chimpanzees with relatively high levels of abnormal behaviours. Both studies 1 and 2 have looked at the effect of passively listening to music and suggest that it has little effect on the behaviour of these chimpanzees. However, in these studies the individuals may have disliked the music, but not wanted to leave an area as they may have been grooming, avoiding other individuals etc., making the cost of avoiding the music relatively high. In the next two studies we allowed individuals to operate devices that enabled them to have choice over whether they listened to music or silence, as choosing to press buttons carries a much lower cost than leaving an area. We aimed to see if when given this finer level of control over their auditory environment whether chimpanzees would show a preference for music, or a specific genre of music, over silence.

## Study three a: National Center for Chimpanzee Care

### Aims and research questions

This study aimed to give the chimpanzees low-cost control over their acoustic environment and provide the opportunity for the chimpanzees to show a preference for classical music, rock music, African folk music or silence. African folk music was included in this study so as to avoid solely investigating the enrichment value of Western music. For this study we created an electronic device that the chimpanzees could interact with to change the sounds. If chimpanzees had a preference for silence or music of a certain type, we expected to see the distribution of button presses to be different from that expected by chance. If they had little interest in music we also expected that the chimpanzees’ motivation to change what was playing would decrease over time.

## Methods

### Study site

The research was undertaken at the National Center for Chimpanzee Care, Michale E. Keeling Center for Comparative Medicine and Research, Department of Veterinary Sciences, The University of Texas M. D. Anderson Cancer Center in Bastrop, Texas.

### Ethics statement

The National Center for Chimpanzee Care is fully accredited by the Association for the Assessment and Accreditation of Laboratory Animal Care-International and approval for study 3a was gained from the Institutional Animal Care and Use Committee (IACUC approval number: 07-92-03887) of University of Texas MD Anderson Cancer Center.

### Participants

A total of 38 subjects, accommodated in four groups (C2: N = 12 adults; 4F; 8M; C4: N = 9 adults and 1 non-adult; 5F; 5M; C5: N = 6 adults; 3F; 3M; C8: N = 7 adults and 1 non-adult; 6F; 2M) ranged in ages from four to 45 years (mean age 28 years; S5 Table). Each group was housed in an enriched outdoor compound with partial visual access of other groups as well as access to indoor dens. Sessions occurred within the indoor area but animals had access to the outside throughout. Music was only broadcast inside and could not be heard outside, ensuring the animals could get away from the music if they wanted to do so.

### Apparatus and procedure

Data were collected during July and August 2006. Sessions lasted one hour and were conducted on Tuesdays and Thursdays, with each group having 16 sessions. The chimpanzees were given a device that could be used to select and listen to classical music, rock music (both different from that used in studies 1 and 2), African folk music or silence (S6 Table). The type of sound could be selected by putting a finger in one of four, vertically arranged holes within a box, three of which were connected to three separate CD players and one hole that turned the music off. The insertion of a finger would activate the photoelectric sensors inside each hole that triggered the playing of the associated music CD or silence. If no further selection had been made after two minutes, the device defaulted back to silence. The vertical order of the sounds within the device changed every four sessions so that each music choice occupied all four holes equally. Testing only began when more than half of the individuals had interacted with the device. No food rewards were used for reinforcing interaction with the device. The type of sound playing at the start of each testing session varied so that each sound was used at the beginning of a session four times. Data were recorded on the frequency and type of choices by a computer attached to the device, video recording was used to identify the number of interactions each individual in the group had with the device and this was summarised by an observer after each session. Unfortunately, as there was no sound associated with the video recording, it was not possible for specific choices to be attributed to a specific individual, meaning that all analyses related to the type of sound selected were group based.

### Data analysis

As the position of the four buttons changed after every four sessions and the outcome of each button was not associated with a visual feature such as colour or pattern, the chimpanzees likely needed the first session in each block of four to understand the new contingencies of the buttons and choices in those first sessions may have been based an understanding of the previous set of contingencies. As such, we removed the first session of each set of four from our analyses examining button choice, leaving 12 sessions. All preference analyses were conducted on the group level as we could not match choices with individuals.

#### Do all chimpanzees across groups have a preference for a specific sound?

For this we ran a Linear Mixed Model (LMM) where the dependent variable was the number of times each button was pressed during each session (log 10 transformed as the original variable was not normally distributed), the independent variable was the sound associated with that button (silence, classical, rock and African folk music) and the random effects were the chimpanzee group (N = 4) and the experiment session number (N = 12). 192 data points came from four groups that each took part in 12 sessions.

#### Does each group have a preference for a specific sound? Do they prefer silence over music?

To identify if each group had a preference for rock, classical, African folk music or silence we compared the distribution of that group’s button presses over the four options with the expected distribution (0.25) using one way Chi squared goodness of fit tests. To see if there was a preference for music over silence we ran binomial tests with an expected frequency of 0.75.

#### Does the interest in pressing the touchscreen decrease over time?

We conducted a Pearson’s correlation to examine the relationship between the session number (N = 16) and the mean number of button presses made by the four groups in each session.

## Results

### Do all chimpanzees across groups have a preference for a specific sound?

**Fig 2:**
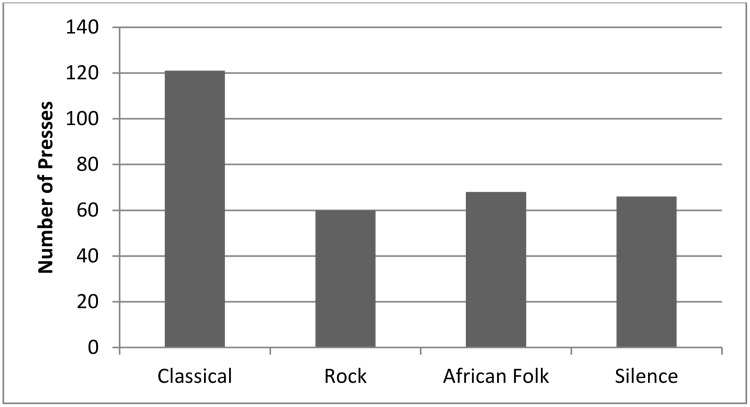
Choices of the four possible button presses made by the four groups across all 64 trials. Despite the higher number of classical presses, there was no significant difference between the different buttons.

An LMM showed that the number of times each button was pressed during each session could not be explained by the sounds associated with different buttons (F(3,188) = 2.19, p = .090; Fig 2) showing there was no overall preference for a specific sound.

### Does each group have a preference for a specific sound? Do they prefer silence over music?

**Fig 3:**
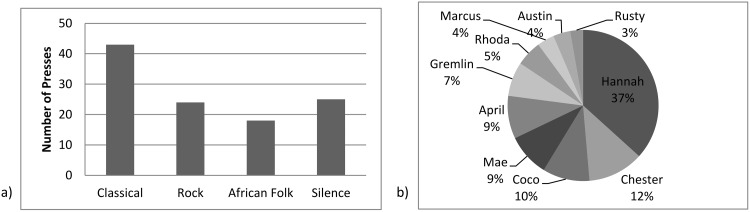
a and b. Graphs showing a) the choices of the four possible button presses made by group C2 and b)–the percentage of total presses by group C2 per individual. Pacer, Cordova and Junie are not included in the graphs as they did not contribute any button presses.

The distribution of Group C2’s button presses was significantly different from the expected distribution (Table 6), with a preference for classical music (Fig 3a). Whether this preference was representative of the 13 individuals in the group, or whether it was driven by a few individuals is unclear. Hannah was responsible for 37% of all of the group’s presses (Fig 3b), although whether she selected classical consistently is unknown. No other groups’ distribution of choices deviated from that expected by chance (Table 6).

**Table 6:** Results of tests investigating preferences for each of the four groups. Significant results are in bold and underlined.

Group	Chi Squared Goodness of Fit for Button Preference	Binomial (0.75) for Preference of Music or Silence
C2	**X**^**2**^**(3) = 11.60, p = .009**	P = .371
C4	X^2^ (3) = 2.88, p = .418	P = .100
C5	X^2^ (3) = 5.65, p = .130	P = .358
C8	X^2^ (3) = 3.20, p = .326	P = .326

### Does the interest in pressing the touchscreen decrease over time?

**Fig 4:**
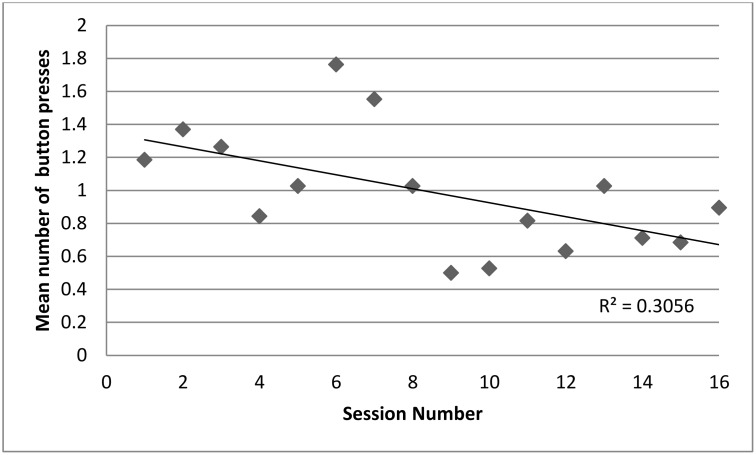
Scatterplot illustrating the mean button presses across all four groups in each of the 16 sessions. Line of best fit is shown.

A Pearson’s correlation (r = -0.55, n = 16, p = .026; Fig 4) showed that there was a significant decrease in button presses over time.

## Discussion

This study shows that, despite having the option to choose the type of sound broadcast and a low cost associated with avoiding a sound they disliked, only one of the four groups (C2) showed a preference for one type of sound, which was classical music and when we looked at all four groups together there was no preference for any of the sounds. The preference of classical music by C2 may not be representative of the whole group as over 75% of button presses were made by just five of the 13 individuals. Recording the type of button pressed by each individual would allow for both group and individual preferences to be established, if they exist.

More strikingly, three of the four groups of chimpanzees did not show a persistent preference for any of the genres of music or silence. The lack of preference for African folk music by any of the four groups indicated that it was not preferred over Western music and that it was unlikely that there is any effect of geographical origin of music on chimpanzee preference. Additionally, all four groups combined showed a decrease in interest in interacting with the device. These findings may result from an indifference to the presence or type of music in their environment, but they may also result from individuals not understanding the contingencies between the buttons and the resulting sound. They may even have been frustrated by the task, which could explain the decrease in interest. Although testing only began when more than half of the individuals had interacted with the device, this did not mean that those individuals understood the contingencies between certain button choices and the sound that subsequently played. To be able to state with more certainty that the animals were indifferent to the presence or type of music we need to know that they had sufficient opportunity to learn how the device worked.

To address these issues we ran another study at Edinburgh Zoo using a touchscreen device, with a training phase and recorded individual choices.

## Study three b: Edinburgh Zoo

### Aims and research questions

This study aimed to continue the work done in study 3a, investigating if when given the choice to control the type of sound a device played, whether the chimpanzees would show any consistent preferences for silence or music. To improve upon the previous study, a new device was created that was able to record the choices made by individuals and a training phase was introduced to increase the chances that the chimpanzees understood the outcome of each button press. African Folk music was not included in this study due to the results of study 3a that suggested that geographical origin of music did not have any effect on chimpanzee preferences.

Unlike studies 1 and 2, this study was conducted in the research pods of Budongo Trail, which were much smaller than the indoor pods music was broadcast into previously. Nevertheless, several individuals were usually present simultaneously in the research pods. Individuals were trained, using food rewards, to press differently patterned buttons on and use a touchscreen to select classical music, pop/rock music or silence. After training was completed there was a period of individual testing, that used rewards to encourage participation, followed by an unrewarded group testing phase that aimed to establish the inherent interest in changing the sounds the device played and the effect of sound button choices on all individuals within the research pods.

This study allowed us to answer the following questions; i) Do chimpanzees prefer music to silence? If individuals had preferences for silence, classical or pop/rock music we expected them to choose the associated button significantly more than expected by chance in both individual and group testing sessions; ii) Is there a difference in the amount of time each individual was exposed to each sound? Based on the finding of study 1, that music did not affect the chimpanzees’ use of space, we predicted that individuals should be exposed to each sound for similar amounts of time; iii) Does the motivation of the chimpanzees to engage with the touchscreen reduce once food rewards are no longer available? As all previous touchscreen research projects conducted with the Edinburgh chimpanzees have used food rewards during testing and training, we predicted that the chimpanzees would become less motivated to interact with the touchscreen once food rewards had been removed, unless listening to certain sounds was intrinsically rewarding; And iv) Do the button choices of third parties affect how long other individuals choose to spend in the research pods? If choices by third parties had adverse effects on individuals in the area, we expected to find a negative relationship between the number of third party sound changes and duration of time in the research pods.

## Methods

### Study site

The training and testing took place in the Research Pods in Budongo Trail covering an area of 26.50m^2^. Access in and out of these pods (connected to the indoor pods by tunnels) was unrestricted during all sessions.

### Apparatus

Stimuli were presented on a 17 inch ELO IntelliTouch touch panel monitor accessible to chimpanzees through a plexiglass testing window. The touch panel was controlled by a customized PC, running Linux Mint. A Bio-Medica Ltd Universal Feeder and pair of speakers were also attached to the computer, while operation of the apparatus was controlled by keyboard, mouse and an additional monitor, which mirrored what was displayed on the touch panel. All experimental programs were written in Python 3 using Kivy libraries.

### Participants

During the testing phase of the project all 18 adults were given the opportunity to participate. If an individual approached the touchscreen and successfully initiated the training session their progress was recorded. Ten individuals never interacted with the touchscreen. One additional chimpanzee started training but did not complete it. Seven individuals completed training but only six of those took part in individual testing. During group testing, all individuals had access to the research pods and could interact with the touchscreen, regardless of their participation in earlier touchscreen training. Six individuals pressed the buttons on the touchscreen during these group sessions. Of these six, four had completed training as well as taking part in individual testing, one had completed training but not taken part in the individual testing and the final individual had not previously interacted with the touchscreen.

### General procedure

Data were collected between January and April 2015. Experimental sessions were run between 09:00 and 10:00 four days a week.

**Fig 5:**
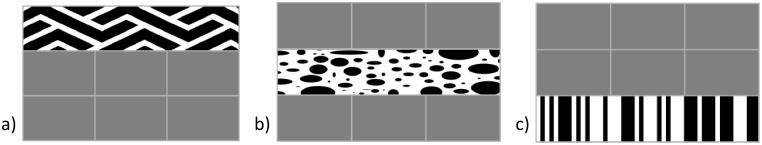
a, b and c. Images of the three touchscreen buttons, as they appeared during training phases. When pressed, each initiated the following actions: (a) turned on classical music for three seconds, (b) turned music off /silence on for three seconds and (c) turned on pop/rock music for three seconds.

The experimental task on the touchscreen consisted of a green start stimulus, a blue holding screen and a choice screen. The choice screen consisted of three equally sized monochrome buttons, each of which had a consistent outcome (striped pattern played pop/rock music for 3 sec, zigzag pattern played classical music for 3 sec and spotted pattern gave 3 sec silence; Fig 5a, 5b and 5c). During training the buttons were the size of a third of the touchscreen to make it easier for the chimpanzees to press the buttons, meaning that there were three possible positions that they randomly appeared in (Fig 5). During testing, when the three buttons were presented simultaneously, the buttons were smaller to increase the diversity of locations the buttons were presented in and to prevent individuals simply being able to keep their finger in the same place and be rewarded for pressing without looking at the pattern of the button. The positions of the buttons during testing were randomly distributed across nine possible positions in each trial.

### Training

**Fig 6:**
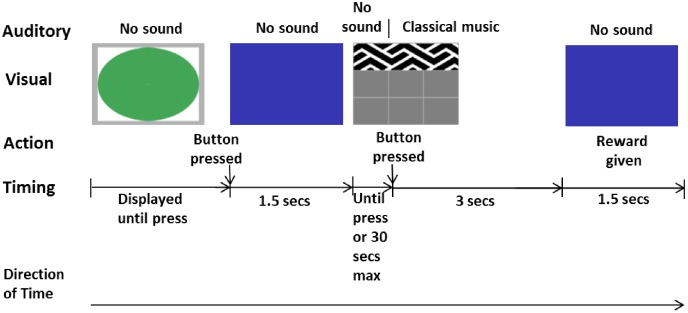
Illustration of the first trial in a classical button training session. This shows the touchscreen images, associated auditory output, actions of the chimpanzees or experimenter, and timings. The downwards arrows indicates a change which is the result of the adjacent action, and is not reflected in an immediate change of visual stimulus. Training continued until the Classical music button had been successfully pressed 10 times, after which the touchscreen was turned off whilst the next training phase was loaded on the computer. If the touchscreen was not interacted with for 30 seconds during a training session, it reverted back to the green circle screen.

There were four levels of training that had to be completed before an individual was able to take part in individual testing: 1) the first type of music button (four individuals started with classical first and three with pop/rock first) was presented singly with the first three seconds of a randomly selected piece of music from a playlist of seven playing when the button was pressed, 2) the other music button presented in the same manner as the previous level, 3) the silence button presented singly along with a randomly selected piece of music out of a choice of 14 (7 classical and 7 pop/rock; S2 Table), which always started at the beginning of the song, so that when the button was pressed the music would stop and there would be silence for three seconds 4) a mixed block with three presentations of each of the three previous levels. Fig 6 shows the order of events within a single training trial and a reward of half a grape was provided when a button was pressed. If an individual did not complete a training level within a single approach of the touchscreen or testing session then the remaining button presses were completed the next time the individual approached the touchscreen, whether it was later in the session or on another day. Once all four levels of training were complete, individual testing could begin.

### Individual testing

**Fig 7:**
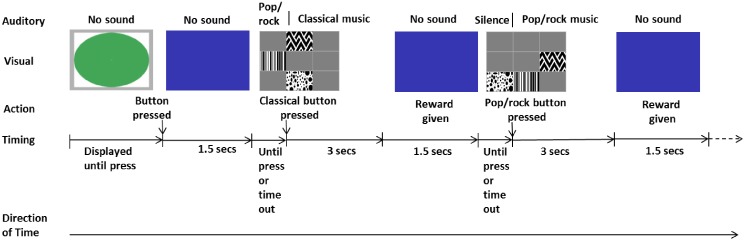
Example first two trials during a session of individual testing. Layout is as described in Fig 6. Testing continued until 40 buttons, not including the green start button, had been successfully pressed. If the touchscreen was not interacted with for 30 seconds, it reverted back to the green circle screen. If an individual did not complete the testing within a single approach of the touchscreen or experimental session then the remaining button presses were completed the next time the individual approached the touchscreen, whether it was later in the session or on another day.

Individual testing began within the group after six individuals had completed at least half of the training stages. Testing trials were broadly similar to training trials (Fig 6), but differed in the following ways: instead of presenting a large single button, all three buttons were presented at once with their position on the screen randomised over the 9 possible presentation locations (Fig 7). Individuals had to complete 40 trials; 10 where the appearance of the buttons on the screen coincided with classical music starting to play, 10 in which buttons appeared with pop/rock music and 20 where no music accompanied the button screen appearing, the order of which was randomised. Frek was the only individual to complete more than 40 trails as he required two experimental sessions to complete the testing and, due to the randomised order of the trials, he had to complete 68 trials in order to have encountered the required distribution across the three types of trials. The same 14 pieces of music were used for individual and group testing as during training (S2 Table) and always started at the beginning of the piece of music. If the button screen appeared with music and the button for the same type of music was selected, three seconds of a new randomly selected piece of music from that playlist would play. All button presses were rewarded to ensure non-differential reinforcement for the three buttons.

### Group testing

To encourage the chimpanzees into the research pods a bale of straw (approximately 10kg) and 7kg of primate pellets were spread across the two pods. As the chimpanzees were let into the research pods the touchscreen was already displaying the three buttons in a randomised position. For three trials classical music was already playing as the individuals entered the pods, for three pop/rock music was playing, for three sessions there was silence and for three sessions the touchscreen was not physically available to the participants and no music was played (total of 12 trials). This sound would continue until a button was pressed or the trial ended after 60 minutes. If an individual approached the touchscreen and pressed a button, the corresponding genre of music would play or the music would be turned off, until a new button was pressed. If no new button was pressed that music or silence would continue until the end of the trial. No rewards were given for pressing the touchscreen during this phase. On pressing a button, the buttons would disappear and the selected music or silence would play for 3 seconds. After that, the blue holding screen would be displayed for 1.5 seconds before starting a new trial, which began with the start stimulus. If the touchscreen was silent and the ‘off’/silence button was pressed the silence would continue but if one of the types of music was playing and the same button was pressed a new randomly selected piece of music from the same playlist would begin playing. Data was collected on the number and type of buttons pressed by each individual and how long individuals were present in the pod.

### Observational data collection

Observations were recorded simultaneously by two observers at different vantage points using a Panasonic SDR-S26 video camera and an Olympus DM-650 Dictaphone. The times of all entries into and exits out of the Research Pods were recorded as well as all approaches to the touchscreen. An approach was defined as an individual coming within 20 cm of the touchscreen and staying in front of it for more than five seconds, with their face directed towards the touchscreen. An approach was considered terminated as soon as the individual turned their face away from the touchscreen or started moving away from it. The start and end time of all approaches were recorded, as well as if any buttons were pressed, what type of button was pressed and how many times.

A second coder was used to confirm the start and end time of approaches from video footage. This was used to compare the number of approaches within three randomly selected trials. An Interobserver reliability test was run giving a Kappa value of .959 where p < .001, indicating that this behaviour had been reliably recorded.

## Data analysis

### In individual and group testing situations, do individuals have a preference for a specific sound? Do they prefer silence over music?

We performed individual level analysis and ran these tests for each of the 6 individuals who completed training and the individual testing. To identify if an individual had a preference for either pop/rock, classical music or silence we compared the distribution of an individual’s button presses over the 3 options with the expected distribution (.33) using one way chi squared goodness of fit tests. To see if they had a preference for music over silence we ran binomial tests with an expected frequency of 0.66. For individual testing these tests were run for each of the 6 individuals who completed training and the individual testing (N = 6). For group testing only one individual was included in the analysis for the one way chi squared goodness of fit tests as chi squared tests cannot be run with less than five expected values in each cell. Two individuals were included in the binomial tests as they had more than three button presses.

### Is there a difference in the amount of time each individual was exposed to each sound?

For this we ran an LMM where the dependent variable was how long an individual was exposed to each sound during each stay in the research pods, the independent variable was the type of sound (silence, pop/rock, classical), and the random effects were individual identity and the experiment session number. There was a total of 398 data points from 17 individuals that voluntarily entered the research pods during the course of the nine sessions where the touchscreen was active.

### Is the duration of time spent in the research pods dependent upon the number of times the sound is changed by third party individuals?

To test this we ran a LMM where the dependent variable was the length of time of each stay in the research pods by an individual, the independent variable was how many times the sound was changed by another individual pressing a button during that stay and the random effects were individual identity and the experiment session number to control for these factors. We only included stays in the research pods where another individual pressed a button or buttons to see the effect of the sound being changed by third party individuals. Data was analysed on 196 pod entries from 17 individuals that voluntarily entered the research pods for a period including at least one button press by a third party during the course of the nine sessions where the touchscreen was active.

### Does the interest in approaching or touching the touchscreen decrease over time?

We examined whether interest in the touchscreen, amongst those who chose to approach or interact with it changed with time. For approaches, we calculated the group rate for approaches in each session (total number of approaches by the 12 individuals who had approached the touchscreen at least once divided by the total duration all 12 individuals spent in the pod). We used a Kendall’s Tau, due to the small sample size, to see if the rate of approaches changed over the course of the nine sessions where the touchscreen was in use. We then used a Paired T-test to compare the individual rates (N = 12) for the first three sessions with the last three. We then replicated these analyses for button presses, with data being taken from the N = 6 individuals who pressed the touchscreen buttons in the group sessions.

## Results

### In individual and group testing situations, do individuals have a preference for a specific sound? Do they prefer silence over music?

**Fig 8:**
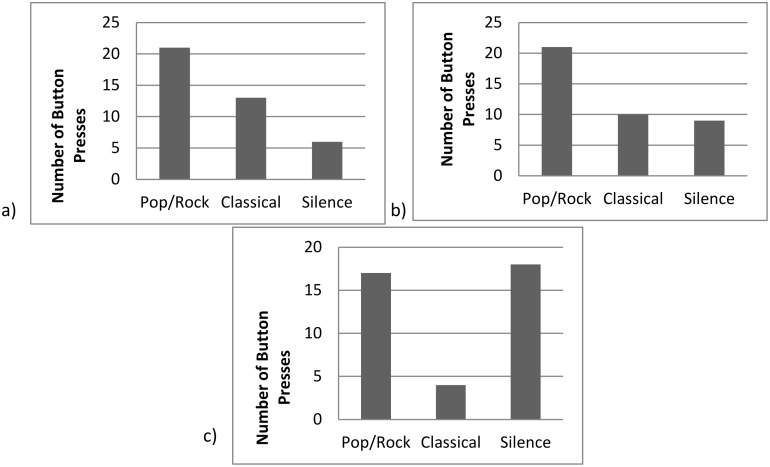
a, b and c. The number of times a) Edith, b) Pearl and c) Kilimi pressed each type of button during individual testing.

During individual testing, Edith showed a preference for music over silence and pop/rock music over classical music or silence (Table 7; Fig 8a) but did not show any preferences during group testing. Similarly, Pearl showed a preference for pop/rock music over classical music or silence (Table 7; Fig 8b) and no preferences during group testing. Kilimi showed a preference for both pop/rock music and silence over classical music and a trend for a preference for music over silence (Table 7; Fig 8c) but no group testing preferences. Frek showed a preference for music over silence during individual testing (Table 7) but did not use the touchscreen during group testing. Eva and Louis (Table 7) did not show any preferences. Louis completed training but then did not use the touchscreen during group testing. Cindy completed training but did not participate in individual testing and Emma did not complete training. Although Cindy and Emma did use the touchscreen during the group testing period, they did not have enough presses for statistical analysis to be conducted (Cindy: Silence = 2 Class = 1 Pop = 1; Emma: Silence = 1 Class = 0 Pop = 2).

**Table 7:** Results of tests investigating preferences for Edith, Eva, Pearl, Kilimi, Louis and Frek. Trends are italicised and significant results are shown in bold and underlined.

Individual	Testing Phase (number of presses)	Chi Squared Goodness of Fit for Button Preference	Binomial (0.66) for Preference of Music or Silence
Edith	Individual Testing (Silence = 6 Class = 13 Pop = 21)	**(X**^**2**^**(2) = 8.45, *p* = .015) Preference for pop/rock**	**P = .009 Preference for Music (34/40) over Silence (6/40)**
	Group Testing (Silence = 9 Class = 7 Pop = 6)	(X^2^ (2) = 0.64, *p* = .727) No Preference	P = .634 No Preference
Eva	Individual Testing (Silence = 19 Class = 14 Pop = 19)	(X^2^ (2) = 0.96, *p* = .618) No Preference	P = .341 No Preference
	Group Testing (Pop = 1)	N/A	N/A
Pearl	Individual Testing (Silence = 9 Class = 10 Pop = 21)	**(X**^**2**^**(2) = 6.65, *p* = .036) Preference for pop/rock**	p = .082No Preference
	Group Testing (Silence = 1 Class = 1 Pop = 1)	N/A	N/A
Kilimi	Individual Testing (Silence = 18 Class = 4 Pop = 17)	**(X**^**2**^**(2) = 9.39, *p* = .009) Preference for silence and pop/rock over classical**	*P* = .*060 Trend for Preference of Music (21/40) over Silence (18/40)*
	Group Testing (Silence = 3 Class = 3 Pop = 5)	N/A	P = .227 No Preference
Louis	Individual Testing (Silence = 14 Class = 10 Pop = 16)	(X^2^ (2) = 1.40, *p* = .497) No Preference	P = .452 No Preference
	Group Testing (Did not participate)	N/A	N/A
Frek	Individual Testing (Silence = 14 Class = 21 Pop = 27)	(X^2^ (2) = 3.35, *p* = .187) No Preference	**P = .040 Preference for Music (48/62) over Silence (14/62)**
	Group Testing (Did not participate)	N/A	N/A

### Is there a difference in the amount of time each individual was exposed to each sound?

There was no difference in the amount of time individuals were exposed to each of the sounds (F(2,394) = 1.05, p = .352).

### Is the duration of time spent in the research pods dependent upon the number of times the sound is changed?

**Fig 9:**
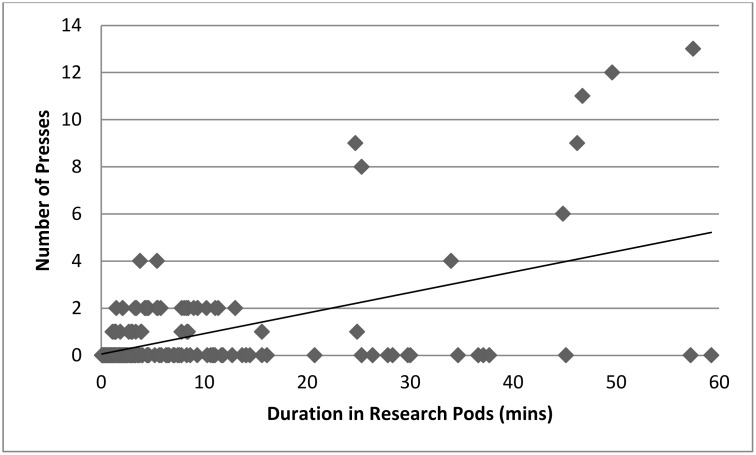
Relationship between how long the chimpanzees stayed in the research pods and the number of third party presses. Scatterplot with line of best fit. Each data point represents a distinct stay in the research pods by one of the 17 individuals.

There was a significant effect of the number of times the sound changes on the length of time spent by an individual in the research pods (F(1,193) = 89.53 p = < .001, N = 17). Fig 9 shows that as length of time spent in pod increases, so do number of third party presses. If presses by a third party were having a negative effect upon the others in the pod then a negative correlation was expected.

### Does the interest in approaching and pressing the touchscreen decrease over time of exposure?

The chimpanzees did not change how often they approached the touchscreen across the nine active sessions (*τ*_b_ = 0.23, *n* = 9, *p* = .399) or between the first three and last three sessions (t(9) = -1.09, p = .306). The total number of presses in each session decreased across the nine active sessions, but this association was not significant (*τ*_b_ = -0.22, *n* = 9, *p* = .404). Equally when examining the presses of the 6 individuals to interact with the touchscreen during group testing there was no difference in the mean number of presses in the first three and last three sessions (t(6) = -1.07, p = .285). Table 8 shows the number of times each individual pressed buttons during each session.

**Table 8:** The number of times each individual pressed buttons during each of the nine experimental sessions. The total number of button presses across all nine sessions is 45 and it can be seen that, with the exception of the first session, engagement with the touchscreen was low.

Session Number	1	2	3	5	6	7	9	11	12	Total number of button presses per Individual
Edith	12	0	4	0	0	1	2	3	0	22
Eva	0	0	0	2	0	0	0	0	0	2
Pearl	1	0	1	0	0	0	0	0	1	3
Emma	0	0	0	0	0	1	2	0	0	3
Kilimi	5	0	2	0	2	2	0	0	0	11
Cindy	0	0	0	0	0	4	0	0	0	4
Total number of button presses per session	18	0	7	2	2	6	4	3	1	45

## Discussion

This study shows that when the chimpanzees were given the option to learn about the touchscreen device and the outcome of the actions, they did not show any consistent preference for music or silence that lasted over both individual and group trials, which supports the results of study 3a. The existence of some individual preferences during the individual testing, (e.g. Pearl showed a preference for pop/rock and Frek displayed a preference for music over silence), may have been actual preferences, but as these preferences did not persist into group testing they may have been an artefact of individual reinforcement patterns during individual testing.

Once rewards were removed during group testing, motivation to engage with the touchscreen was low (Table 8), particularly after the first session (where rewards were likely expected based on the previous individual testing trials). It is possible that the presses that did occur during group testing were showing genuine preferences, but they occurred at such low levels that we did not have a sufficient number of data points to be able to detect these preferences.

The apparent lack of consistent preferences could also be due to the individuals not understanding the task. Despite having to complete four training phases before individual testing could begin, the animals may not have fully understood the relationship between the visual stimuli (the different buttons) and the auditory stimuli (the different sounds). In particular, even if individuals had a basic understanding of this relationship, the three second exposure to the different sounds after pressing the button may not have been sufficient for them to distinguish between the types of music.

In line with the findings from Study 1, there was no significant difference in the amount of time the animals were exposed to each sound condition in the group testing sessions. Thus it seems they did not leave the research pods to limit their exposure to any sounds they did not like. We also investigated whether there was a negative relationship between the number of changes to the sounds playing and duration of time spent in the research pods, to check whether exposure to repeated third party sound switches may have had negative effects on group members. We felt it was important to test this, as even if the individuals interacting with the device found it enriching, it was possible this was at the detriment of other group members affected by the broadcast sounds. However, the relationship that was found was positive, meaning that the longer an individual was in the research pods the more times there were changes of sound condition. This shows that having a third party changing between the sounds did not cause them to leave the research pods.

## General discussion

The results of these four studies show that the presence of music has very limited effects on how chimpanzees use the space within their enclosure or the expression of behaviour and that they do not show a consistent preference for either music or silence. We present convergent evidence from four studies over two research sites that have examined responses of chimpanzees at both group and individual levels to passive listening and active choice paradigms. This is the first project to include all of these aspects when investigating the effect of music on chimpanzee welfare.

The fact that music had little overall effect on the behaviour of the chimpanzees could have been influenced by relatively low levels of exposure to music over the course of the study. This was an inevitable result of allowing the chimpanzees the option of avoiding the music. During study 1, the mean duration spent in the music pod across 18 individuals during each music period was just over 15 minutes and the average total exposure to music across all of the 38 trials that were included in the analyses was 2.77 hours, or just 14.6% of the total time they could have been exposed to music. Our results suggest that the chimpanzees were not avoiding the music but equally did not seek it out. If it had been possible to play music for several hours a day, as in other zoo-based auditory enrichment studies [15, 16], there would have been a greater chance of an individual being exposed to the music for longer and more chronic exposure music, may then have had a greater effect on behaviour.

However, another possible explanation for our results could be that chimpanzees do not find music enjoyable. This is strongly suggested by our result from study 1 where the individuals displayed significantly less socially active behaviours whilst the music was playing. Ritvo and MacDonald [16] found one of three orangutans given the choice of listening to music or silence had no preference for either and that all three animals were unable to distinguish music from samples of scrambled non-music, suggesting that not only is music something primates do not find enriching, it is something they potentially perceive in the same manner as noise. It is maybe unsurprising that non-human primates do not respond to music positively due to music being a human construct. Music seems to be universal amongst human populations [25] and it is even suggested that human language evolved from vocal origins in the form of communal singing [26]. However, what constitutes music varies greatly between cultures [25] and therefore it may be unlikely that a human construct with global variation will be considered enjoyable by any other species, even one as closely related as chimpanzees. A recent fMRI study [27] discovered an area of the human auditory cortex, which is selectively active in response to music rather than speech, regardless of genre, instrumentation or personal enjoyment of the music played. The authors question if this type of organisation is present in the brains of other species or whether this area of selective processing of music is a derived, uniquely human trait. If this is lacking in chimpanzees, and other primates, it could explain why music is something they seem indifferent towards.

Whilst it is possible that music is something that is not appreciated by any species other than humans, the fact that the results of our studies contrast with existing work [11, 12, 15] highlights the need for further investigation in different species and contexts. We have shown that group-housed chimpanzees do not appear to benefit from the presence of music but this does not necessarily mean that this is the case for all non-human primate species, especially those that are not group-housed.

In conclusion, our studies suggest that it is doubtful that either classical or contemporary pop/rock music have any positive enriching effects for group-housed captive chimpanzees. We suggest that despite the ease and cost efficiency of playing music as a form of enrichment, this is not an effective strategy and alternative types of enrichment should be employed. If facilities play music for the enjoyment of the care staff, music with less than 90 BPM should be played preferentially, but as long as the chimpanzees have the opportunity to avoid the music, as they did in these studies, it is unlikely to have any profoundly negative effects on behaviour.

## Supporting information

S1 Fig Speaker inside the insulated box. (DOCX)

S1 Table Demographic information on chimpanzees at Edinburgh Zoo. (DOCX)

S2 Table Music used in studies 1, 2 and 3b. ‘We are Young’ by Fun ft Janelle Monáe was not used during study 3b.
(DOCX)

S3 Table Playlists of music used in studies 1 and 2. (DOCX)

S4 Table Mean observation times for three focal individuals during study 2. (DOCX)

S5 Table Demographic information on four groups of chimpanzees at National Centre for Chimpanzee Care. (DOCX)

S6 Table Music used in study 3a. (DOCX)

S1 Dataset Datasets for studies 1, 2, 3a and 3b. (XLSX)
