# Supplementary figures and images for: Is music enriching for group-housed captive chimpanzees (Pan troglodytes)?
Source: PLoS One. 2017 Mar 29;12(3):e0172672. doi: 10.1371/journal.pone.0172672 (PMC5371285; doi:10.1371/journal.pone.0172672)

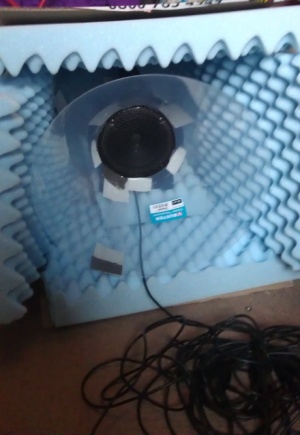

Supplement: S1 Fig — (DOCX) [file pone.0172672.s001.docx]
