# Supplementary material for: Is music enriching for group-housed captive chimpanzees (Pan troglodytes)?
Source: PLoS One. 2017 Mar 29;12(3):e0172672. doi: 10.1371/journal.pone.0172672 (PMC5371285; doi:10.1371/journal.pone.0172672)
Supplement: S1 Table — (DOCX) [file pone.0172672.s002.docx]

| Name | ID | Sex | Age at Start of Study | Rearing | Original Group |
| --- | --- | --- | --- | --- | --- |
| Cindy | CI | F | 49 | Wild | Edinburgh |
| David | DA | M | 38 | Mother | Edinburgh |
| Edith | ED | F | 16 | Mother | Beekes Bergen |
| Emma | EM | F | 33 | Mother | Edinburgh |
| Eva | EV | F | 34 | Nursery | Beekes Bergen |
| Frek | FR | M | 19 | Mother | Beekes Bergen |
| Heleen | HL | F | 21 | Mother | Beekes Bergen |
| Kilimi | KL | F | 20 | Mother | Edinburgh |
| Kindia | KD | M | 16 | Mother | Edinburgh |
| Lianne | LI | F | 24 | Mother | Beekes Bergen |
| Liberius | LB | M | 14 | Mother | Edinburgh |
| Louis | LO | M | 37 | Wild | Edinburgh |
| Lucy | LU | F | 36 | Mother | Edinburgh |
| Paul | PA | M | 19 | Mother | Beekes Bergen |
| Pearl | PE | F | 44 | Wild | Beekes Bergen |
| Qafzeh | Q | M | 21 | Mother | Edinburgh |
| Rene | RE | M | 20 | Nursery | Beekes Bergen |
| Sophie | SO | F | 33 | Nursery | Beekes Bergen |
