# Supplementary material for: Is music enriching for group-housed captive chimpanzees (Pan troglodytes)?
Source: PLoS One. 2017 Mar 29;12(3):e0172672. doi: 10.1371/journal.pone.0172672 (PMC5371285; doi:10.1371/journal.pone.0172672)
Supplement: S2 Table — ‘We are Young’ by Fun ft Janelle Monáe was not used during study 3b. (DOCX) [file pone.0172672.s003.docx]

| Classical |  | Pop/rock |  |
| --- | --- | --- | --- |
| Title (Beats per minute) | Composer/Performed by (Duration mins:seconds) | Title (Beats per minute) | Artist (Duration mins:seconds) |
| Serenade in B Flat, Gran Partita – Adagio (68) | Mozart/Academy of St Martin in the Fields, conducted by Sir Neville Marriner (5:31) | Too Close (128) | Alex Clare (3:44) |
| Brandenburg Concerto #2 In G, BWV 1048 - 2. Andante (62) | JS Bach/Chamber Orchestra of Europe (3:21) | Rollin‘ in the Deep (100) | Adele (3:48) |
| Nocturne for piano No. 16 in E flat major, Op. 55/2, B. 152/2 (66) | Chopin/Daniel Barenboim (3:23) | Locked out of Heaven (144) | Bruno Mars (3:54) |
| BGN (46) | Elgar/ (2:42) | Troublemaker (108) | Olly Murs ft Flo Rida (3:06) |
| Clarinet Concerto in A - Adagio (84) | Mozart/Emma Johnson and the Royal Philharmonic Orchestra (7:31) | One More Night (95) | Maroon 5 (3:40) |
| Maid with the Flaxen Hair (66) | Richard Stoltzman/Slovak Radio Symphony Orchestra (2:49) | ET (92) | Katy Perry ft Kanye West (3:51) |
| Piano Sonata No 14 in C sharp minor Op 27 No 2 Moonlight - Adagio sostenuto (52) | Beethoven/ (5:07) | Beauty and a Beat (132) | Justin Beiber ft Nicki Minaj (3:48) |
|  |  | We are Young (120) | Fun ft Janelle Monáe (4:11) |
