# Supplementary material for: Is music enriching for group-housed captive chimpanzees (Pan troglodytes)?
Source: PLoS One. 2017 Mar 29;12(3):e0172672. doi: 10.1371/journal.pone.0172672 (PMC5371285; doi:10.1371/journal.pone.0172672)
Supplement: S3 Table — (DOCX) [file pone.0172672.s004.docx]

| Classical One | Classical Two | Classical Three | Pop/rock A | Pop/rock B | Pop/rock C |
| --- | --- | --- | --- | --- | --- |
| Serenade in B Flat, Gran Partita – Adagio | Clarinet Concerto in A - Adagio | Piano Sonata No 14 in C sharp minor Op 27 No 2 Moonlight - Adagio sostenuto | Too Close | We are Young | One More Night |
| Nocturne for piano No. 16 in E flat major, Op. 55/2, B. 152/2 | Maid with the Flaxen Hair | Brandenburg Concerto #2 In G, BWV 1048 - 2. Andante | Rollin’ in the Deep | Beauty and a Beat | Troublemaker |
| BGN | Piano Sonata No 14 in C sharp minor Op 27 No 2 Moonlight - Adagio sostenuto | Serenade in B Flat, Gran Partita – Adagio | Locked out of Heaven | ET | Too Close |
| Clarinet Concerto in A - Adagio | Brandenburg Concerto #2 In G, BWV 1048 - 2. Andante | Nocturne for piano No. 16 in E flat major, Op. 55/2, B. 152/2 | We are Young | One More Night | Rollin’ in the Deep |
| Maid with the Flaxen Hair | Serenade in B Flat, Gran Partita – Adagio | BGN | Beauty and a Beat | Troublemaker | Locked out of Heaven |
| Piano Sonata No 14 in C sharp minor Op 27 No 2 Moonlight - Adagio sostenuto | Nocturne for piano No. 16 in E flat major, Op. 55/2, B. 152/2 | Clarinet Concerto in A - Adagio | ET | Too Close | We are Young |
| Brandenburg Concerto #2 In G, BWV 1048 - 2. Andante | BGN | Maid with the Flaxen Hair | One More Night | Rollin’ in the Deep | Beauty and a Beat |
|  |  |  | Troublemaker | Locked out of Heaven | ET |
