# Supplementary material for: Is music enriching for group-housed captive chimpanzees (Pan troglodytes)?
Source: PLoS One. 2017 Mar 29;12(3):e0172672. doi: 10.1371/journal.pone.0172672 (PMC5371285; doi:10.1371/journal.pone.0172672)
Supplement: S4 Table — (DOCX) [file pone.0172672.s005.docx]

| Focal Individual | First Silence Control Mean in mins (SD) | Music Mean in mins (SD) | Second Silence Control Mean in mins (SD) |
| --- | --- | --- | --- |
| Lianne | 29.40 (4.95) | 27.76 (5.55) | 29.39 (1.20) |
| Paul | 28.31 (2.64) | 19.56 (10.90) | 28.50 (5.00) |
| Rene | 26.73 (4.37) | 22.97 (10.13) | 26.62 (7.01) |
