# Supplementary material for: Is music enriching for group-housed captive chimpanzees (Pan troglodytes)?
Source: PLoS One. 2017 Mar 29;12(3):e0172672. doi: 10.1371/journal.pone.0172672 (PMC5371285; doi:10.1371/journal.pone.0172672)
Supplement: S5 Table — (DOCX) [file pone.0172672.s006.docx]

| Name | Group | Gender | Age at Start of Study | Rearing |
| --- | --- | --- | --- | --- |
| Hannah | C2 | F | 15 | Mother |
| Coco | C2 | F | 21 | Mother |
| June | C2 | F | 40 | Unknown |
| Mae | C2 | F | 41 | Unknown |
| Rhoda | C2 | M | 43 | Unknown |
| Chester | C2 | M | 10 | Mother |
| Rusty | C2 | M | 10 | Mother |
| Austin | C2 | M | 14 | Mother |
| Marcus | C2 | M | 14 | Mother |
| Cordova | C2 | M | 34 | Unknown |
| Gremlin | C2 | M | 35 | Unknown |
| Pacer | C2 | M | 35 | Unknown |
| Cassie | C4 | F | 17 | Mother |
| Emily | C4 | F | 21 | Mother |
| Lulu | C4 | F | 24 | Mother |
| Abbey | C4 | F | 41 | Unknown |
| Sandy | C4 | F | 44 | Unknown |
| Lyle | C4 | M | 7 | Mother |
| Tony | C4 | M | 16 | Mother |
| Doyle | C4 | M | 23 | Mother |
| Punch | C4 | M | 23 | Mother |
| Kudzu | C4 | M | 23 | Mother |
| Misty | C5 | F | 22 | Mother |
| Helga | C5 | F | 40 | Unknown |
| Ursula | C5 | F | 42 | Unknown |
| Cody | C5 | M | 16 | Mother |
| Joey | C5 | M | 34 | Unknown |
| Zippy | C5 | M | 35 | Unknown |
| Zoe | C8 | F | 4 | Mother |
| Cecelia | C8 | F | 15 | Mother |
| Tinker | C8 | F | 22 | Mother |
| Kelley | C8 | F | 40 | Unknown |
| Martha | C8 | F | 40 | Unknown |
| Mary | C8 | F | 41 | Unknown |
| Huey | C8 | M | 15 | Mother |
| Pierre | C8 | M | 44 | Unknown |
