# Supplementary material for: Is music enriching for group-housed captive chimpanzees (Pan troglodytes)?
Source: PLoS One. 2017 Mar 29;12(3):e0172672. doi: 10.1371/journal.pone.0172672 (PMC5371285; doi:10.1371/journal.pone.0172672)
Supplement: S6 Table — (DOCX) [file pone.0172672.s007.docx]

| Genre | CD Used – Title and Artist |
| --- | --- |
| Classical | 25 Classical Favourites - Various composers |
| Rock | Best of 80’s Metal, Vol. 1 - Various artists |
| African Folk | Dance My Children, Dance - Samite |
